# Identification of novel prognostic biomarkers in renal cell carcinoma

**DOI:** 10.18632/aging.104131

**Published:** 2020-11-21

**Authors:** Yuanzhang Zou, Qiu Lu, Qin Yao, Di Dong, Binghai Chen

**Affiliations:** 1 Department of Urology, Affiliated Hospital of Jiangsu University, Jiangsu University, Zhenjiang, Jiangsu, China

**Keywords:** prognostic signature, WDR72, renal cell carcinoma, OS, DFS

## Abstract

Objective: To identify novel prognostic biomarkers in renal cell carcinoma (RCC).

Results: 12 coding genes and one miRNA were finally identified as prognostic biomarkers. All of them were related to a poor prognosis. Lower expression levels of the coding genes were observed in higher clinical stages. Prognostic signatures including 7 biomarkers were identified. Patients in the high-risk group had worse survival than those in the low-risk group. The areas under the curves in different years indicated that it was a valuable signature in prognosis. It was found that elevated WDR72 inhibited the survival and invasion of 786-O and 769P cells *in vitro*.

Conclusions: Thirteen prognostic biomarkers of RCC were identified. Among them, 7 biomarkers comprised a signature to evaluate the RCC prognosis. WDR72 was a cancer suppressor and a potential therapeutic target in RCC.

Methods: Differentially expressed genes/miRNAs (DEGs/DEMs) and prognosis-related genes/miRNAs were acquired from public database. Prognostic biomarkers were identified by overlapping the significant DEGs/DEMs and prognosis-related genes/miRNAs. The associations between these biomarkers and the clinical stages were analyzed. All of these prognostic biomarkers were further investigated with multi-variable Cox regression. Finally, the inhibitory effect of WDR72 on the growth and invasion of RCC cells was studied.

## INTRODUCTION

Renal cancer is one of the most common malignancies that threatens people’s health [1]. Renal clear cell carcinoma (usually called RCC, known as KIRC in databases) makes up the vast majority of kidney cancers and approximately one-third of patients are diagnosed in advanced stages [2, 3]. Furthermore, up to 40% of patients with local RCC ultimately develop metastasis after surgical resections. Antineoplastic drugs are the conventional strategies for cancer treatment [4, 5]. The current therapies for RCC are still unsatisfactory, particularly in those patients with advanced RCC or distant metastasis [6]. Although cancer vaccines have been developed and the preclinical trials of vaccines have shown potential in improving overall survival (OS) of RCC, their use requires extensive additional research [7, 8]. Therefore, it is of vital importance to identify promising biomarkers in RCC.

A large number of genes, including protein-coding genes and noncoding RNAs, are aberrantly expressed during the tumorigenesis and development of carcinoma. Thus, these genes are potential therapeutic targets and biomarkers in tumor management. For instance, Sun et al. [9] showed that CHIP, a protein-coding gene, was markedly downregulated in RCC samples compared with para-cancerous tissues, and its dysregulation was related to the development and prognosis of RCC patients. Therefore, CHIP was considered as a target for RCC treatment and as a biomarker for the prognostication of RCC patients. Similarly, Xu et al. [10] revealed that ISG20 was abnormally expressed in RCC samples and could function as a biomarker and therapeutic target. In addition, with the development of detection technology, similar findings for lncRNA, microRNA, and pseudogenes were reported in numerous studies [11–13].

Currently, high-throughput platforms provide an efficient way to acquire gene expression arrays to identify the differentially expressed genes (DEGs) between cancer and para-cancerous tissues. Researchers can therefore identify novel targets and biomarkers for the treatment of carcinoma [14–17]. The Gene Expression Omnibus (GEO) is a free public database that provides public array and sequence-based functional genomics data. Based on the gene expression arrays from the GEO database, many DEGs have been identified as indicators for early diagnosis and biomarkers for the prognosis of various tumors including RCC [18, 19]. Gene Expression Profiling Interactive Analysis (GEPIA), a novel public database, is a tool used to analyze the RNA-sequencing data from over 10 thousand tumors and normal samples via a standardized procedure [20]. It has been widely used with other databases to explore promising biomarkers and therapeutic targets in various cancers [21–23]. The overlapped genes are likely to be very promising and critical because they are demonstrated in all of the databases.

In this study, we combined the GEO and GEPIA2 databases to identify more valuable prognostic biomarkers in RCC. As expected, we finally identified 13 genes including 12 protein-coding genes and hsa-miR-21-5p as prognostic biomarkers. Among them, 7 genes were identified as new prognostic biomarkers in RCC. Moreover, WDR72 was found to be the most significant prognostic gene in RCC. Based on further study of WDR72, we found that WDR72 was of vital importance in the development of RCC.

## RESULTS

### Identification of DEGs, DEMs and prognostic biomarkers in RCC

**Figure 1:**
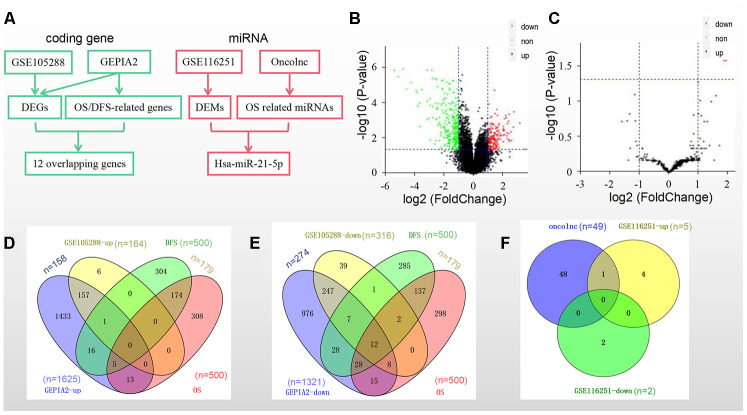
**DEGs/DEMs and prognosis-related genes/miRNAs identification in different datasets and databases.** (**A**) A standardized procedure for the identification of prognostic biomarkers; (**B**) Volcano plot of DEGs from GSE105288; (**C**) Volcano plot of DEMs from GSE116251; (**D**) Intersection of the upregulated genes and (**E**) downregulated genes from the GSE105288 dataset and the GEPIA2 database; (**F**) Intersection of 49 prognostic miRNAs from the Oncolnc database and 7 DEMs from the GSE116251 dataset.

We identified prognostic biomarkers by a standardized procedure (Figure 1A). The data in the GSE105288 dataset showed that there were a total of 480 DEGs in the RCC samples, including 164 upregulated DEGs and 316 downregulated DEGs (Figure 1B), while there were only 5 upregulated DEMs and 2 downregulated DEMs in the GSE116251 dataset (Figure 1C). Moreover, we obtained 1625 upregulated DEGs and 1321 downregulated DEGs in RCC tissues by using GEPIA2 database (Figure 1D and 1E).

The top 500 genes (Supplementary Table 1) that were related to overall survival (OS) and disease-free survival (DFS) separately in GEPIA2 were then screened. Among them were 179 genes related to both OS and DFS (Figure 1D and 1E).

The overlapped genes in both GSE105288 and the above 179 genes in GEPIA2 included 12 ones as follows: WDR72, ALDH6A1, CDS1, SLC25A4, MTURN, ERBB2, NR3C2, OGDHL, BSPRY, HADH, DNASE1L3 and CLDN10 (Figure 1E).

Furthermore, as for the miRNAs OS profile a total of 448 miRNAs were downloaded from the Oncolnc platform. There were 49 miRNAs significantly related to OS (Figure 1F). Hsa-miR-21-5p was the only overlapped miRNA in both DEMs and among the above 49 miRNAs (Figure 1F).

Taken together, the 12 genes and hsa-miR-21-5p were selected as prognostic biomarkers in RCC.

### Gene Ontology (GO) and Kyoto Encyclopedia of Genes and Genomes (KEGG) pathway enrichment

**Figure 2:**
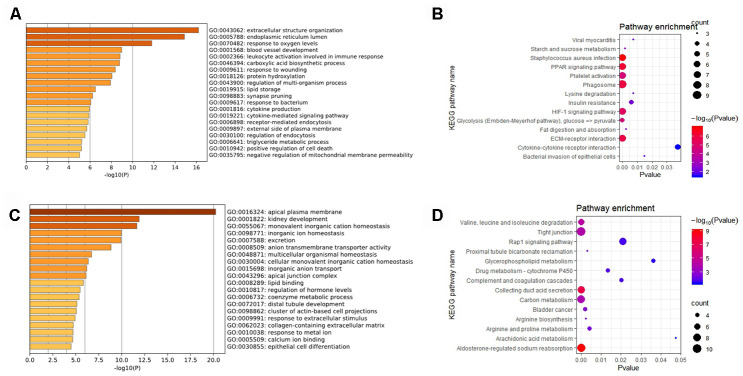
**Results of DEGs for the GO and KEGG pathway analysis.** (**A**) The enriched GO biological processes and (**B**) KEGG pathways of the significant upregulated DEGs; (**C**) The enriched GO biological processes and (**D**) the KEGG pathways of significant downregulated DEGs.

To understand the underlying functions of the significant DEGs, Metascape was used for functional enrichment. The outcomes of GO analysis suggested that “extracellular structure organization”, “endoplasmic reticulum lumen”, and “response to oxygen levels” were the most important items for the upregulated significant DEGs (Figure 2A), while for the downregulated significant DEGs they were “apical plasma membrane”, “kidney development”, and “monovalent inorganic cation homeostasis” (Figure 2C). Moreover, in DEGs analyzed in the KEGG pathway analysis, the DEGs were involved in multiple pathways, including “Staphylococcus aureus infection”, “PPAR signaling pathway”, “Phagosome”, “Aldosterone-regulated sodium reabsorption”, and the “Collecting duct acid secretion” pathways (Figure 2B and 2D). These pathways may provide researchers with directions of further mechanistic investigations of these DEGs in RCC.

### Hsa-miR-21-5p expression was negatively related to 11 of the coding genes in RCC

**Figure 3:**
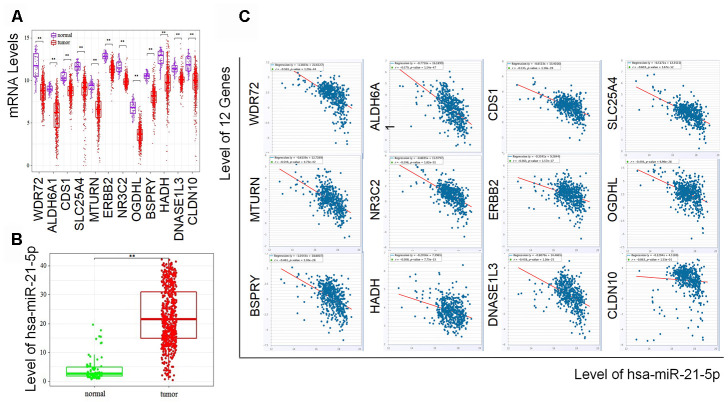
**Expressionss of the prognostic biomarkers and their coexpression in RCC.** (**A**) The 12 coding genes were downregulated and (**B**) hsa-miR-21-5p was upregulated in tumor samples compared with normal samples; (**C**) Showing the coexpression of prognostic coding genes and hsa-miR-21-5p in RCC, among which 11 coding genes (WDR72, ALDH6A1, CDS1, SLC25A4, MTURN, ERBB2, NR3C2, OGDHL, BSPRY, HADH, DNASE1L3) were negatively related to the expression of hsa-miR-21-5p. CLDN10 was the only gene with a P value over 0.05.

To further study the association between hsa-miR-21-5p and the 12 coding genes in RCC, we compared their levels between tumor and normal samples from UCSC Xena as well as the TCGA database. Consistent with the results of the GSE105288 and GSE116251 datasets, the levels of all 12 coding genes were lower in the RCC samples (Figure 3A), while the expression of hsa-miR-21-5p was significantly increased (Figure 3B). To figure out the expression relationship between hsa-miR-21-5p and the 12 coding genes, we next checked the coexpression of hsa-miR-21-5p and the 12 genes in StarBase. The results indicated that hsa-miR-21-5p was negatively related to these coding genes except for CLDN10 (Figure 3C), which was the only gene with a P value over 0.05.

### Decreased level of the 12 coding genes and increased level of hsa-miR-21-5p showed poorer prognosis of RCC

**Figure 4:**
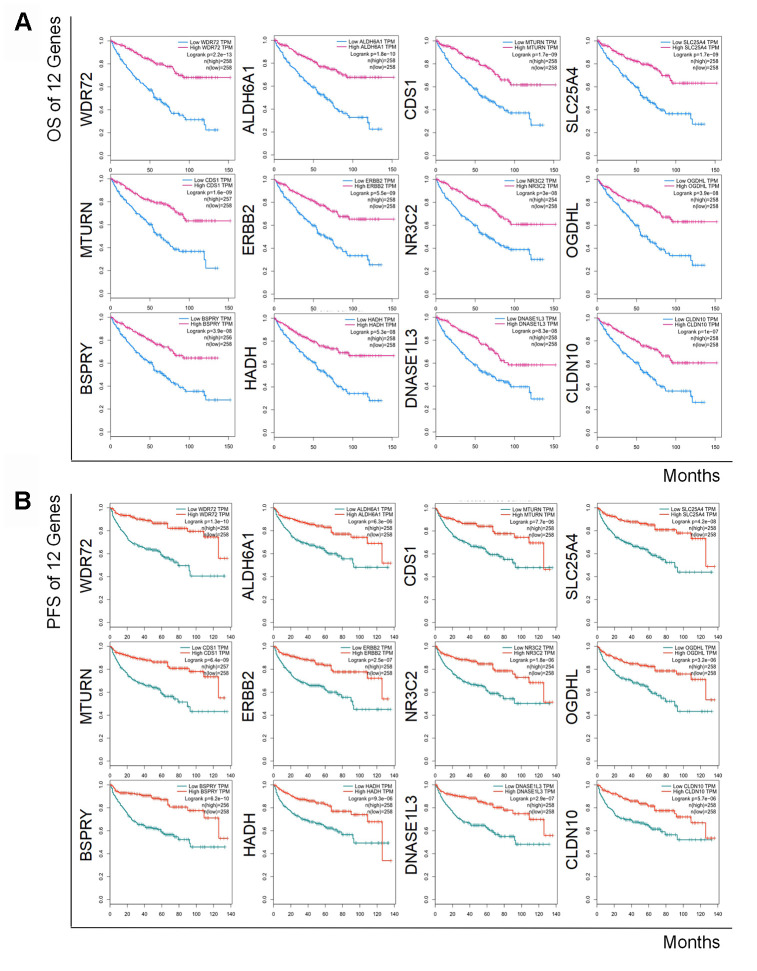
**Kaplan-Meier curves of OS and DFS of 12 prognostic coding genes.** Lower expression of all 12 coding genes was relevant to both unfavorable OS (**A**) and worse DFS (**B**) in patients with RCC.

To determine the exact prognostic significance of the identified genes, we evaluated the OS and DFS of the 12 coding genes as well as the OS of hsa-miR-21-5p in GEPIA2 and Oncolnc, respectively. The results showed that high expression of hsa-miR-21-5p was associated with poor OS and DFS (Supplementary Figure 1), and a decreased level of all 12 coding genes indicated a poor OS and DFS (Figure 4A and 4B), which suggested that these genes and hsa-miR-21-5p could indeed act as biomarkers in the prognostication of RCC.

### The prognostic biomarkers were related to the clinical stages of RCC

**Figure 5:**
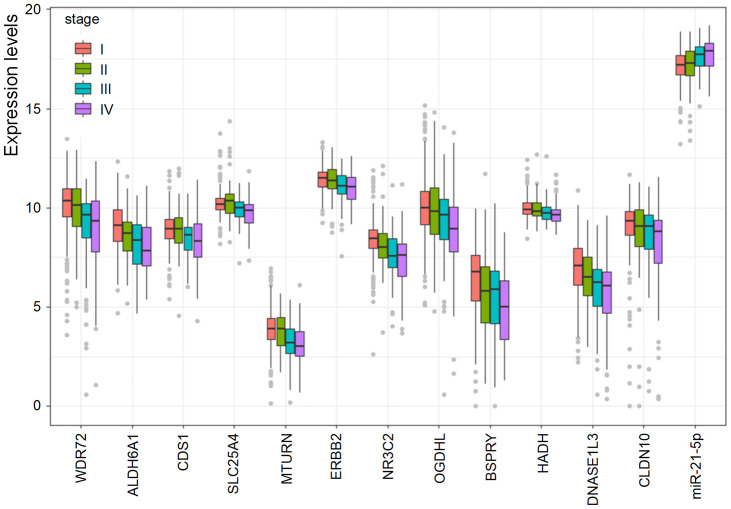
**The association of the prognostic biomarkers and clinical stages.** Lower levels of the 12 coding genes were observed in higher clinical stages. However, the level of hsa-miR-21-5p was elevated in higher clinical stages.

To investigate the association between the prognostic biomarkers and the clinical stages, the levels of the biomarkers at different stages were evaluated. As shown in Figure 5, the levels of the 12 coding genes were lower in higher stages, and some genes, for instance, WDR72, ALDH6A1, OGDHL, and DNASE1L3, were gradually decreased as stage increased. However, increased expression of hsa-miR-21-5p was positively related to increased stage.

### Gene signatures predicted the OS and DFS of RCC

Multivariate Cox regression analysis was used to further explore the signature of the multiple genes regarding the survival of RCC. A group of seven genes (WDR72, ALDH6A1, CDS1, HADH, DNASE1L3, CLDN10, hsa-miR-21-5p) were finally exported into the model as a molecular signature to predict OS (Supplementary Table 2). Five genes (WDR72, ALDH6A1, OGDHL, HADH, and DNASE1L3) exported were found to be a molecular signature to predict DFS (Supplementary Table 3).

**Figure 6:**
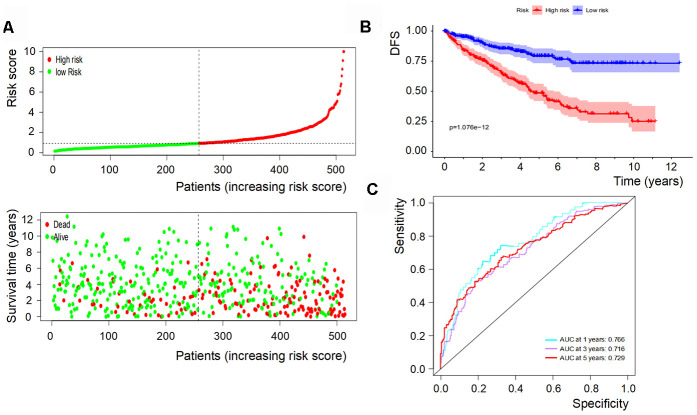
**OS of the signature with multiple genes in RCC.** (**A**) Patients were classified by risk score and their survival status; (**B**) OS of the signature of multiple genes in this model, which indicated that patients in the low-risk group had a more favorable OS than those in the high-risk group. (**C**) ROC curves suggested that the accuracy of this model was 76.6%, 71.6% and 72.9% at 1, 3 and 5 years, respectively.

**Figure 7:**
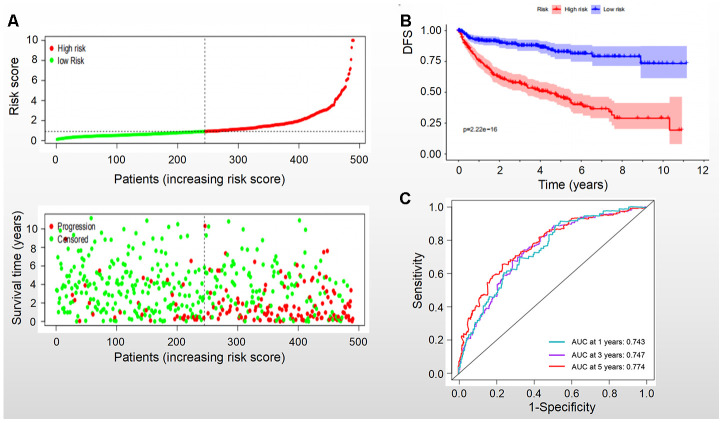
**DFS of the signature with multiple genes in RCC.** (**A**) Patients were classified by risk score and their survival status; (**B**) DFS of the signature of multiple genes in this model, which indicated that patients in the low-risk group had a better DFS than those in the high-risk group. (**C**) ROC curves suggested that the accuracy of this model was 74.3%, 74.7% and 77.4% at 1, 3 and 5 years, respectively.

Based on the levels of the above seven biomarkers in the risk model, all patients were divided into two groups: low- and high-risk groups (Figures 6A, 7A). The patients in the low-risk group had a more favorable OS and DFS than those in the high-risk group (Figure 6B, 7B). The accuracy of this OS model (the area under the ROC curve) was 76.6%, 71.6% and 72.9% at 1, 3 and 5 years, respectively (P<0.001) (Figure 6C). Similarly, the accuracy of this DFS model (the area under the ROC curve) was 74.3%, 74.7% and 77.4% at 1, 3 and 5 years, respectively (P<0.001) (Figure 7C). These data suggested that the signature was quite valuable in predicting OS and DFS of RCC, and it was more convincing in predicting OS than DFS.

### Increased WDR72 expression inhibited the survival and invasion of RCC cell lines *in vitro*

**Figure 8:**
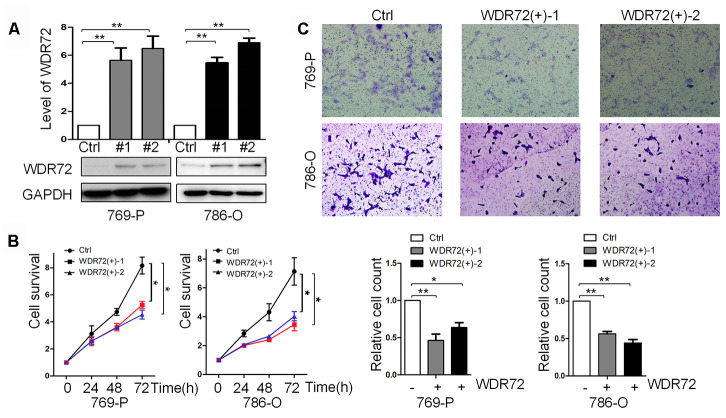
**Role of WDR72 overexpression in cell survival and invasion of RCC.** (**A**) The results of western blot showed that WDR72 was successfully overexpressed in 769-P and 786-O cells; overexpression of WDR72 remarkably decreased the survival (**B**) and invasiveness (**C**) of 769-P and 786-O cells.

WDR72 was found to be the most significant prognostic gene among the identified coding genes in RCC. To further investigate its role in cell proliferation and invasion, WDR72 was successfully overexpressed in RCC 769P (clone #1 and #2) and 786-O (clone #1 and #2) cells by a lentivirus system (Figure 8A). CCK-8 assay displayed overexpression of WDR72 significantly inhibited cell proliferation (Figure 8B). Then, by cell invasion assay, we found that overexpression of WDR72 decreased the invasiveness of RCC cells (Figure 8C). These data suggested that increased expression of WDR72 inhibited the survival and invasion of RCC cell lines. Therefore, WDR72 might serve as a cancer suppressor in RCC.

## Discussion and Conclusion

Recently, the development of high-throughput gene detection technology uncovered big biological data from tumors and provided resources for the identification of promising therapeutic targets, diagnostic and prognostic biomarkers in cancers [16]. In addition, the development of databases that integrate RNA-seq data and clinical information of various tumor types offers an available approach to explore biomarkers in cancers. Several databases for tumors have been developed [24–26]. In the present study, we used the GSE105288 dataset and the GEPIA2 database to acquire 432 significant DEGs. Functional enrichment analysis revealed that these DEGs were involved in “apical plasma membrane”, “kidney development”, “monovalent inorganic cation homeostasis”, “Staphylococcus aureus infection”, “PPAR signaling pathway”, “Phagosome”, etc. Among these functions and pathways, “apical plasma membrane” is important for renal function regulation [27], while “Staphylococcus aureus infection, PPAR signaling pathway, and phagosome” are key pathways in RCC development [28–30].

By overlapping the significant DEGs and the prognostic genes, 12 prognostic coding genes, including WDR72, ALDH6A1, CDS1, SLC25A4, MTURN, ERBB2, NR3C2, OGDHL, BSPRY, HADH, DNASE1L3, and CLDN10 were finally identified. Similarly, a prognostic miRNA gene was identified by using the GSE116251 dataset and the Oncolnc database. These 13 genes were considered as prognostic biomarkers for RCC. Among them, 3 coding genes have been previously reported to be involved in the biological process of RCC. Lu et al. [31] detected the expression of ALDH6A1 in 50 pairs of clinical samples. ALDH6A1 was expressed at lower levels in the vast majority of tumor samples compared with the corresponding normal tissues. In addition, lower expression of ALDH6A1was related to a worse prognosis of RCC. *In vitro* results demonstrated that ALDH6A1 overexpression could decrease cell proliferation and migration, but its regulatory mechanism was not uncovered.

Some other examples are NR3C2 [32] and HADH [33]. It was shown that lower expression of NR3C2 was related to a worse OS and progression-free survival of RCC. NR3C2 functions as a tumor suppressor both *in vitro* and *in vivo*. Consistent with these studies, we also found that the expression levels of ALDH6A1, NR3C2, and HADH were downregulated. Moreover, we found another 9 coding genes that were also down-regulated and associated with worse OS and DFS of RCC patients. These genes could be novel candidates for prognostic biomarkers in RCC.

We further explored the association between these candidate biomarkers and the clinical stages of patients with RCC. It was found that some biomarkers showed a significant correlation with the stages, suggesting these biomarkers may also be valuable in staging of RCC patients.

The combinatorial effect of these prognostic indicators was investigated in our study. A signature of seven genes was obtained and a risk model was constructed based on the signature for predicting the survival of patients with RCC. The signature could accurately classify the patients into low-risk and high-risk groups, which had different OS and DFS outcomes.

Among these biomarkers, WDR72 was the most significant one for the prognosis of RCC. The previous literature revealed that WDR72 has mainly been investigated in amelogenesis imperfecta, and has rarely been reported in malignancies [34–36]. The previous studies of WDR72 were in bladder and esophageal cancer. WDR72 was suggested to be a candidate biomarker for identifying the risk of recurrence. In addition, WDR72 could also be used as an indicator in the diagnosis of esophageal cancer [37, 38]. In the present study, we first demonstrated that WDR72, as a novel gene, was involved in the development of RCC. The outcomes *in vitro* showed that overexpression of WDR72 decreased the survival and invasiveness of RCC cells, which was in accordance with the results from the database.

There were also nonoverlapping genes. For instance, the expression level of ABCC4, a nonoverlapped gene, is elevated in RCC and it is required in the regulation of cell survival [39]. ABCC4 induces cell arrest and apoptosis. However, it is unknown whether ABCC4 is valuable in the prognosis of RCC. In contrast, WDR72 is one of the most significant prognostic genes in RCC. Low expression of WDR72 indicates a worse OS and DFS. The data obtained about WDR72 *in vitro* also provides additional evidence that WDR72 is a potential tumor suppressor and therapeutic target in RCC.

As for the standard approach to RCC's prognosis, we believe that it is much stricter when using WDR72. WDR72 was screened from a database including 9,736 tumors and 8,587 normal samples. The P values of OS and DFS were 1.31E-10 and 2.25E-13 respectively. These data suggest that WDR72 is likely to be a cancer suppressor and a novel target for RCC treatment.

Furthermore, we found that hsa-miR-21-5p was overexpressed and correlated with an unfavorable prognosis of RCC, which is another candidate biomarker consistent with previous studies [40, 41]. In addition, we found that the expression of hsa-miR-21-5p was inversely related to the investigated coding genes (except for CLDN10), which indicated that hsa-miR-21-5p might target these 11 genes involved in the development of RCC. Hence, the regulatory mechanism of these prognostic biomarkers may be via a ceRNA modulating pattern, which has been well known in miRNA-mRNA regulation [42, 43].

In conclusion, we identified 13 genes as prognostic biomarkers in renal cell carcinoma and revealed their probable regulatory mechanism in the progression of RCC. Among them, WDR72 may also be a novel therapeutic target for RCC. The molecular mechanism of this biomarker in RCC deserves further validation and other candidate biomarkers should be further investigated in the future.

## MATERIALS AND METHODS

### Differential gene expression analysis

The gene expression profile of GSE105288 [44] and the miRNA expression profile of GSE116251 [45] were downloaded from the GEO database (https://www.ncbi.nlm.nih.gov/geo). The GSE105288 dataset includes 35 RCC samples and 9 normal renal samples. GSE116251 is comprised of 18 RCC tissues and 18 normal renal tissues. The comparison was performed in GEO2R to identify DEGs and differentially expressed miRNAs (DEMs). Since the adj. P-value could amend the false positive rate, we selected the adj. P-value< 0.05 and |log_2_FC|>1 as cut-off criteria. In addition, with q-value < 0.01 and |log_2_FC|>1, the DEGs of RCC in the Gene Expression Profiling Interactive Analysis (GEPIA2) database were also downloaded using the ANOVA method. The DEGs from the GSE105288 dataset and the GEPIA2 database were considered as the significant DEGs, while the DEMs from GSE116251 were defined as significant DEMs. GEPIA2 is an open web version for visual analysis based on the RNA sequencing expression data of 9,736 tumors and 8,587 normal samples from the Cancer Genome Atlas (TCGA) and the GTEx projects [46]. GEO2R (https://https://www.ncbi.nlm.nih.gov/geo/geo2r), is an interactive web tool to identify DEGs through comparing grouped samples based on the GEO query and R-packages.

### Functional and pathway enrichment analysis

Metascape is an efficient platform for gene function annotation analysis [47]. It is composed of functional enrichment, interactome investigation, genomic annotation and packages approximately 50 independent knowledge-bases into a single platform. Significant DEGs were input into the Metascape program to carry out GO and KEGG pathway analysis. *Homo sapiens* was selected and the P-value <0.05 was considered statistically significant.

### Prognosis-related genes and miRNAs screening

Both the most significant OS-related genes and DFS-related genes in RCC were screened in the GEPIA2 database and the top 500 genes with P-value<0.05 were downloaded. Oncolnc (http://www.oncolnc.org) is an online program containing survival data for 8647 patients of 21 cancer types from the TCGA database. The clinical and expression data of the mRNAs, microRNAs, and long non-coding RNAs can be directly downloaded from Oncolnc. Thus, we downloaded the miRNAs OS profile of RCC and the BH-adjusted p-value <0.05 was thought to indicate prognosis-related miRNAs.

### Prognostic biomarkers identification

The significant DEGs and significant prognosis-related overlapped genes were used to construct prognostic biomarkers in RCC. Similarly, the significant DEMs and prognosis-related miRNAs were also used to establish prognostic biomarkers in RCC. OS was defined as the time from treatment of RCC to death. Death could be either cancer-related or cancer-unrelated. DFS was defined as time from treatment of RCC to recurrence. A recurrence was considered as the development of RCC confirmed by pathological or radiological diagnosis at the operative site, in the regional lymph nodes, or a distant metastasis.

### Verification of expression and prognostic significance of the prognostic biomarkers

UCSC Xena (https://xena.ucsc.edu/) is a visual exploration resource for both data downloading and online analysis based on Xena Browser. It also allows biologists to figure out the relationships between genomic and clinical data. StarBase (http://starbase.sysu.edu.cn/index.php) is a tool for exploring noncoding RNA functions and regulatory mechanisms. StarBase identifies millions of interactions among miRNA, ncRNA and mRNA using multiple dimensional data. The mRNA expression data of prognostic genes and prognostic miRNAs were downloaded from the UCSC Xena database and the TCGA database (https://www.cancer.gov/tcga), respectively. The prognostic significance of prognostic genes and miRNA were evaluated in the GEPIA2 and Oncolnc platforms separately. Furthermore, we analyzed the coexpression of the prognostic genes and prognostic miRNA in RCC with the StarBase version 3 database.

### Association between prognostic biomarkers and clinical stage

We further analyzed the association between the expression of these prognostic biomarkers and the clinical stage of RCC patients. The expression levels of all the 13 biomarkers were compared in different stages (I, II, III, and IV). Differences between every two stages were analyzed by *t*-test. The ggplot2 package of R software (4.0.2) was used for result visualization. *P* value <0.05 was considered statistically significant.

### Multivariate Cox regression analysis

To understand the combinatorial effect of the identified biomarkers, multivariate Cox regression analysis was performed and a risk model was established. Patients were divided into high-risk and low-risk groups by the cut-off value (1.0) of the risk score. Then, the survival of the two groups was evaluated by Kaplan-Meier survival curves with log-rank test. Time-dependent ROC curves were performed to assess the performance of the signature classifier at 1, 3 and 5 years.

### Cell culture

The HEK293T, 786-O and 769P cell lines were purchased from the American Type Culture Collection (ATCC). The HEK293T, 786-O and 769-P cells were respectively cultured in RPM1-DMEM medium (with L-glutamine) and RPM1-1640 medium (with L-glutamine) (Fcmacs Biotech, Nanjing, China) in a 5% CO_2_ incubator at 37° C. Both kinds of medium were complete medium supplemented with 10% fetal bovine serum (FBS) (Fcmacs Biotech) and 1% penicillin and streptomycin (Sangon, Shanghai, China).

### Gene overexpression

Lenti-sgRNA^WDR72^ was constructed by lenti sgRNA (MS2). The sgRNA^WDR72^ plasmid was mixed with the REV, GAG and VSVG plasmids (Hanheng Biotech, Shanghai, China) to construct Lenti-sgRNA^WDR72^. After transfection for 48 h, recombinant lentiviruses were obtained. Lenti-dCas9 and Lenti-MPH were constructed in the same way. The 786-O and 769-P cells were infected by the Lenti-dCas9 and Lenti-MPH to generate 769P^dCas9+MPH+^ and 786-O^dCas9+MPH+^ cells, which were finally infected with Lenti-sgRNA^WDR72^. The 1640 medium containing puromycin was used to screen the cells for 3-4 days. The cells with stably overexpressed WDR72 were selected for further study. We also validated the expression of WDR72 by western blot.

### Western blot

Radioimmunoprecipitation assay (RIPA) solution was utilized to extract the total protein of the cells and BCA reagent (Beyotime, Shanghai, China) was used to determine their concentration. Protein samples were separated by electrophoresis and then transferred to polyvinylidene difluoride membranes (Millipore, USA) for 2 h. Subsequently, the membranes were blocked with 3% bovine serum albumin (Sangon, Shanghai, China) and incubated with anti-WDR72 primary antibody (Cell Signaling, Danvers, MA, USA) at 4° C overnight. The next day, 1×TBST was used to wash the membranes 3 times. Then, a secondary antibody was used to incubate the membranes at room temperature for 1.5 h. Finally, the bands were scanned by a gel-imaging platform (UVP, CA, USA).

### Cell proliferation assay

According to the instructions of the manufacturer, the Cell Counting Kit-8 (CCK-8) (Vazyme, Nanjing, China) assay was used to measure cell proliferation. Experimental cells were inoculated in the 96-well plate (each well with 1 × 10^4^ cells) and cultured in an incubator for 24 h, 48 h, and 72 h. Then, 10% CCK-8 reagent was added into each well and continuously cultured for 1 h. The optical density (*OD*) at 450 nm was measured. Three independent experiments were carried out.

### Cell invasion assay

Invasion assay was conducted using 24-well Transwell chambers (Corning Incorporated, USA). 786-O^WDR72(+)^, 769-P^WDR72(+)^ cells as well as the control were seeded in the upper chamber (2×10^4^ cells) with 500 μL 1640 medium. Then, the cells were cultured in the incubator for 24 h. After wiping away the noninvading cells on the surface of the upper chamber, we stained the cells in the lower chamber with crystal violet (BBI life sciences, Shanghai, China) at room temperature for 30 min. The chamber was photographed before the cells were counted. Five independent areas of each well were acquired and the mean number of cells was used for graphing.

### Statistical analysis

Every experiment was repeated no less than 3 times. The comparisons of different groups were performed with independent sample *t*-test. A two-sided *P* value<0.05 was defined as statistically significant. GraphPad prism 8.0 and R software (4.0.2) were used for graphing.

## Supplementary Material

Supplementary Figure 1

Supplementary Table 1

Supplementary Tables 2 and 3
