# Supplementary figures and images for: Identification of novel prognostic biomarkers in renal cell carcinoma
Source: Aging (Albany NY). 2020 Nov 21;12(24):25304–18. doi: 10.18632/aging.104131 (PMC7803519; doi:10.18632/aging.104131)

SUPPLEMENTARY FIGURE

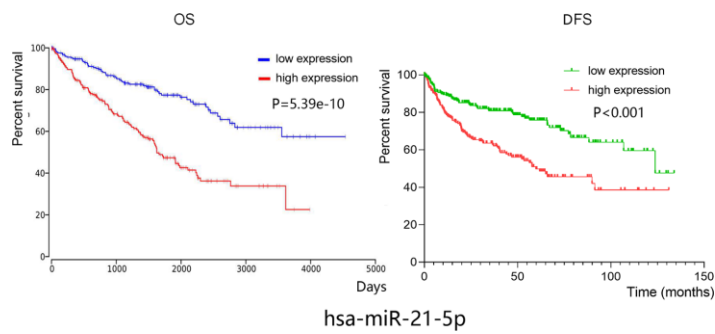

Supplementary Figure 1. Kaplan-Meier curves of OS and DFS of hsa-miR-21-5p in RCC.

Supplement: Supplementary Figure 1 [file aging-12-104131-s001.pdf]
