# Supplementary material for: Identification of novel prognostic biomarkers in renal cell carcinoma
Source: Aging (Albany NY). 2020 Nov 21;12(24):25304–18. doi: 10.18632/aging.104131 (PMC7803519; doi:10.18632/aging.104131)
Supplement: Supplementary Tables 2 and 3 [file aging-12-104131-s003.pdf]

SUPPLEMENTARY TABLES

Supplementary Table 2. Multivariate Cox regression analysis of pronostic biomarkers for OS.

Gene	Multivariate Cox regression analysis			
	**HR**	**Low 95% CI**	**High 95% CI**	**Coefficient**
WDR72	0.924252937	0.835673516	1.022221567	-0.078769504
ALDH6A1	0.841058328	0.722946199	0.978467156	-0.173094266
CDS1	1.206009676	0.992697351	1.465158881	0.187317122
HADH	0.553366621	0.385647718	0.794026783	-0.59173453
DNASE1L3	0.859011757	0.76805078	0.960745329	-0.15197267
CLDN10	0.890757465	0.808626404	0.981230464	-0.115683094
miR-21-5p	1.237694323	0.931886916	1.64385529	0.213250232

Supplementary Table 3. Multivariate Cox regression analysis of pronostic biomarkers for DFS.

Gene	Multivariate Cox regression analysis			
	**HR**	**Low 95% CI**	**High 95% CI**	**Coefficient**
WDR72	0.913106585	0.830464779	1.003972302	-0.090902664
ALDH6A1	0.827531871	0.712829082	0.960691721	0.827531871
OGDHL	0.933006303	0.853832	1.019522296	0.933006303
HADH	0.674380721	0.469142544	0.969405487	0.674380721
DNASE1L3	0.800762037	0.718979677	0.891846961	0.800762037
